# Freeze-dried *Lactobacillus plantarum* 299v increases iron absorption in young females—Double isotope sequential single-blind studies in menstruating women

**DOI:** 10.1371/journal.pone.0189141

**Published:** 2017-12-13

**Authors:** Michael Hoppe, Gunilla Önning, Lena Hulthén

**Affiliations:** 1 Department of Gastroenterology and Hepatology, Section for Clinical Nutrition, Sahlgrenska University Hospital, Gothenburg, Sweden; 2 Department of Internal Medicine and Clinical Nutrition, Sahlgrenska Academy at the University of Gothenburg, Gothenburg, Sweden; 3 Biomedical Nutrition, Pure and Applied Biochemistry, Center for Applied Life Sciences, Lund University, Lund, Sweden; 4 Probi AB, Ideon Gamma 1, Lund, Sweden; TNO, NETHERLANDS

## Abstract

**Background:**

The probiotic strain *Lactobacillus plantarum* 299v has earlier been shown to increase iron absorption when added to foods. However, it is not known if the same probiotic strain in a freeze-dried format included in a capsule increases the iron absorption.

**Objective:**

The aim of this study was to test the hypotheses that non-heme iron absorption from a light meal is promoted by a simultaneous intake of freeze-dried *Lactobacillus plantarum* 299v (Lp299v, DSM 9843).

**Study design:**

With a single blinded placebo controlled sequential design, iron absorption from a light breakfast meal administered with or without capsules containing 10^10^ cfu freeze-dried Lp299v was studied in healthy female volunteers of fertile age. The methodology used was a double isotope technique (^59^Fe and ^55^Fe). Two studies were performed using the same protocol.

**Results:**

In study 1, the absorption of iron from a meal without Lp299v was found to be 17.4 ± 13.4%, and from an identical meal with Lp299v was found to be 22.4 ± 17.3% (mean ± SD). This difference was statistically significant (*p* = 0.040, n = 14).

In study 2, the absorption of iron from a meal without Lp299v was found to be 20.9 ± 13.1%, and from an identical meal with Lp299v found to be 24.5 ± 12.0% (mean ± SD, n = 28), which again was statistically significant (*p* = 0.003).

**Conclusion:**

Freeze-dried Lp299v enhances the absorption of iron when administered together with a meal with a high iron bioavailability.

**Trial registration:**

ClinicalTrials.gov Identifier: NCT02131870

## Introduction

Iron deficiency is the most common nutrient deficiency in the world, and is primarily present in women of reproductive age, who are at higher risk of developing iron deficiency because of iron losses during menstruation [1]. Other groups at risk of developing iron deficiency are infants, young children, adolescents, elderly with a poor nutrient intake, vegetarians, athletes and pregnant women [1–4]. Anemia affects an estimated two billion people, and causes approximately 0.8 million deaths a year worldwide [5]. Although iron deficiency is not the only cause of anemia, the WHO estimates that iron deficiency is responsible for approximately 50% of all anemia cases [6]. This approximates to 1 billion cases of iron-deficiency anemia worldwide. Consequences of iron deficiency, and primarily iron deficiency anemia, are diversified and range from fatigue [7,8] and decreased aerobic performance [9–12], to altered cognitive functions [13,14], immune system alterations [15] and increased risk of maternal and child mortality [16–18].

In order to improve iron status by dietary means, iron deficient individuals are recommended to eat foods rich in iron or in combination with food components that increase the bioavailability of the dietary iron, where the latter has shown to be of major importance [19]. Ascorbic acid [20] and meat [21] are examples of dietary factors that have shown to enhance non-heme iron absorption, whereas calcium [22], polyphenols [23] and phytic acid [24] inhibit non-heme iron absorption. Another factor that may increase the absorption of iron is probiotics [25,26]. The definition of probiotics is, according to FAO/WHO, "*live micro-organisms which*, *when administered in adequate amounts*, *confer a health benefit on the host*" [27]. The human gastrointestinal (GI) tract hosts an astounding amazing >100 trillion (10^14^) microbes. In the human colon more than 1200 bacterial species have been identified, with each healthy individual harboring at least 160 shared species [28]. In iron deficient and anaemic women in southern India it was observed that the amount of faecal lactobacilli was significantly lower than in a control group but there was no difference between the two groups with respect to other investigated bacteria [29]. Earlier human meal studies have also shown that intake of lactic acid fermented vegetables and cereals give a significant increase in iron absorption [30–32]. An intake of a lactobacilli may therefore increase the iron absorption and this has been investigated in earlier trials using the strain *Lactobacillus plantarum* 299v (Lp299v). Lp299v has been shown to possess the ability to survive the passage through the gastrointestinal tract [33], to colonize human intestinal mucosa [34,35], and also to increase the absorption of iron [25,26] when present live in fermented oat gruel and in fruit drinks.

The use of probiotics in food products poses some challenges when it comes to shelf-life, especially at room temperature. One potential alternative is to use freeze-dried lactobacilli in a capsule, with a shelf-life of 24 months. However, it is not known if freeze dried probiotics included in a capsule will increase the iron absorption similar to what has been shown for probiotics added directly into foods. The working hypothesis of the present study was that non-heme iron absorption from a meal is promoted by a simultaneous intake of freeze-dried *Lactobacillus plantarum* 299v (Lp299v).

## Material and methods

### General protocol / design

The study was executed by a nutritionist and a research engineer at the laboratory of the Department of Internal Medicine and Clinical Nutrition, Sahlgrenska Academy at the University of Gothenburg, Sweden. The study was designed as a single blinded placebo controlled sequential iron absorption trial in healthy, Swedish female volunteers of reproductive age. The subjects were students recruited at two Swedish universities, Sahlgrenska Academy at the University of Gothenburg, and Chalmers University of Technology. The methodology used was a double isotope technique [26,36,37], where iron absorption from two different meals (A and B) is determined by labeling the meals with either ^55^Fe or ^59^Fe. One of the benefits of this technique is that each subject becomes her own control. The double isotope technique can be considered the present golden standard in iron absorption methodology. With the double isotope technique iron absorption is assessed by calculating the difference between the administered radioactivity and the radioactivity measured either in blood (iron incorporation in erythrocytes) or in the total body (in a whole body counter). When using the whole body counting the iron absorption from the ^59^Fe (a γ-emitting isotope) labeled meal is calculated as the percentage of detected whole-body radioactivity, corrected for physical decay and background radioactivity. However, absorption from ^55^Fe, which is a β-emitting isotope, cannot be detected by whole-body counting. Thus, after WBC, a blood sample is drawn in which the relative absorption of each of the two isotopes is determined using a liquid scintillator. This relative absorption is then used to calculate the total body ^55^Fe absorption.

A light meal, consisting of two breakfast buns with table margarine and orange marmalade, and a glass of water, were served on four consecutive mornings to fasting subjects. During the first two mornings, together with the light meal, the fasting subjects were served capsules containing no lactobacilli (A), whereas capsules containing 10^10^ colony-forming units (cfu) Lp299v (B) were served on morning 3 and 4. The reason for chosing the order AABB was to avoid any remaining lactobacilli effect that may occur if Lp299v were administered before the placebo capsules [25]. Also, comparative studies have shown that iron absorption from two different meals labeled with either ^55^Fe or ^59^Fe does not differ between individuals regardless of administration order [37]. In order to reduce the day-to-day-variation in iron absorption [38] each meal (i.e. with and without Lp299v) was administered during two consecutive days from which the mean daily iron absorption was calculated. Two separate studies with the same protocol were performed. The number of subjects included in each of the two studies was 18 and 36, respectively. For study 1 participant were recruited during January–February 2014, and the study was executed during February–March 2014. For study 2 participants were recruited during April–May 2014, and the study was executed during May–June 2014. The study was registered at Clinical trials (NCT02131870). The authors confirm that all ongoing and related trials for this intervention are registered.

### Meal content

Each meal consisted of two breakfast buns (made from a total of 80 grams of low extraction wheat flour, 56 grams of water, 5.2 grams of yeast, 2.6 g of sugar, and 0.8 g of NaCl) with Flora table margarine (15 grams), orange marmalade (20 grams) and a glass of water (200 mL). When baking the buns, they were fermented for 2 x 30 minutes, and then baked at 240°C for 10 min. After baking the buns were kept frozen at -20°C and thawed before serving. In addition the meal contained 0.6 mg iron, 21 mg Ca, and 2.6 mg ascorbic acid, the two latter being dietary factors known to influence iron absorption. The phytate content in the buns was negligible since a low extraction wheat flower was used and the dough was fermented twice [31]. The recipe for making the buns / wheat rolls has been used by our research group for almost 40 years and it was specially developed at our lab as to maximize the reduction of phytates [39]. It was also the basis from which the iron absorption algorithm by Hallberg and Hulthén was developed [24]. During the 35 years that the wheat rolls has been used by our research group the same individual in our staff has been involved in baking them. In our experience, there would not be any difference in phytate content in the meals between study 1 and 2.

Together with each meal a total of 3 capsules were administered. The capsules used, provided by Probi AB (Lund, Sweden), are vegetable capsules composed of hydroxypropylmethyl cellulose. The capsules are composed to have a medium dissolution profile and they will be dissolved in stomach after 20–25 minutes.

The capsules in meal A consisted of:

- One capsule containing 30 μg of folic acid, 12 mg of ascorbic acid and 4.2 mg of iron.
- Two capsules, each containing 50 μl ^55^FeCl_3_ in 0.1 M HCl and potato starch.

The capsules in meal B consisted of:

- One capsule containing 30 μg of folic acid, 12 mg of ascorbic acid, and 4.2 mg iron + 10^10^ cfu lyophilised Lp299v.
- Two capsules, each containing 50 μl ^59^FeCl_3_ in 0.1 M HCl and potato starch.

### Measurement of iron absorption

**Fig 1:**
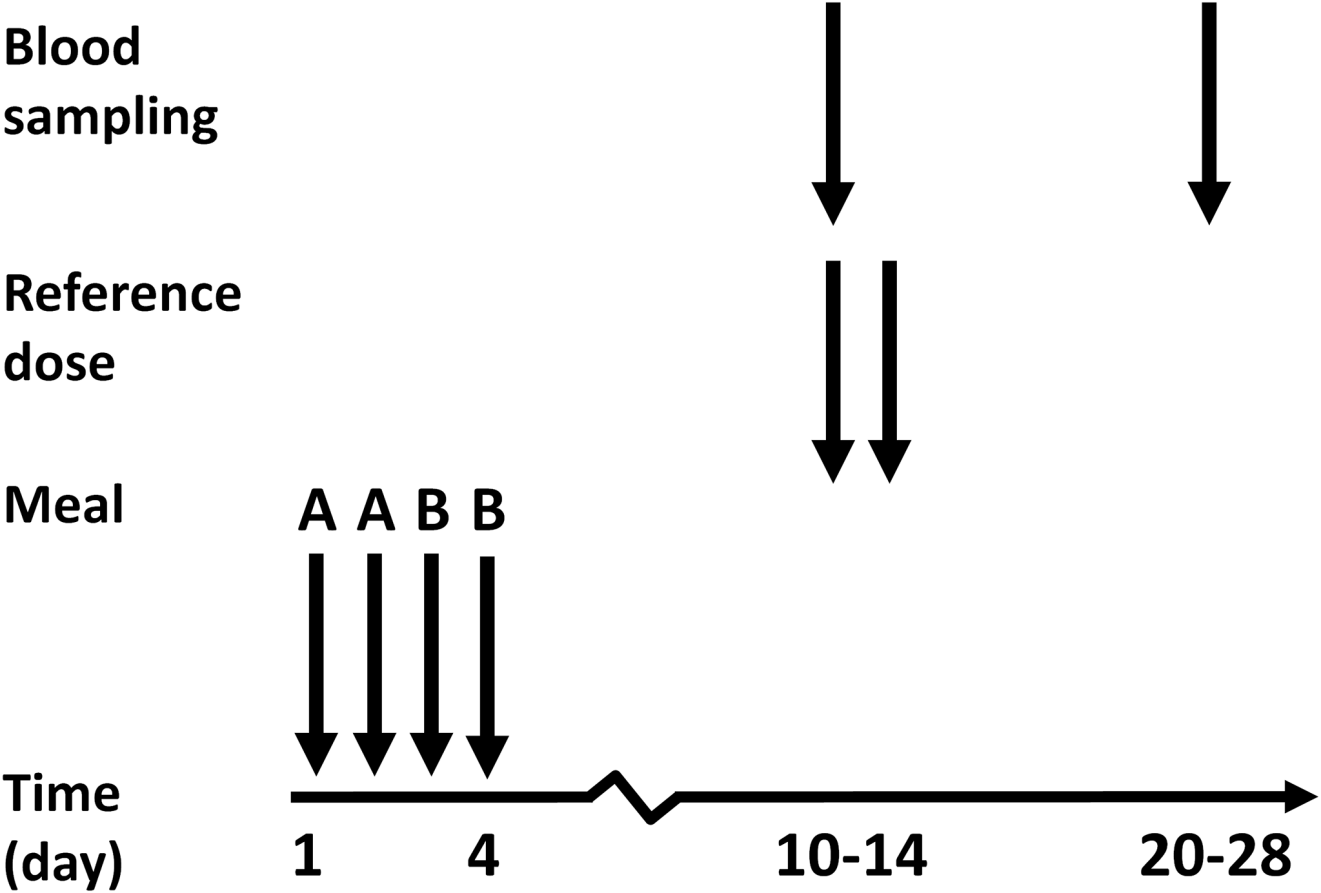
Study design. The iron content in two different meals (A and B) was labeled with either ^55^Fe or ^59^Fe. Meal A contained no lactobacilli and was served on morning 1 and 2, whereas meal B, which contained 10^10^ cfu lyophilised Lp299v, was served on morning 3 and 4. Ten to 14 days after the meals were administered, blood samples were drawn and the radiation from the two Fe isotopes were determined. Directly after drawing the blood sample a reference dose (3 mg ^59^Fe-labeled iron (II) + 30 mg of ascorbic acid dissolved in 10 mL of 0.01 M HCl) was administered. The following morning yet another identical reference dose was served. After a further 10–14 days the daily mean absorption from the two reference doses was analysed in blood samples.

The two capsules labeled with radioisotopes (as FeCl_3_ in HCl) were prepared right before serving. The lower half was filled with 320 mg of potato starch. The radioisotope was added by pippeting, whereupon the capsules upper part was mounted. The capsules were swallowed within 60 seconds from when they were prepared. To maximize isotope exchange [40] between the iron in the capsules and in the meal, the subjects were instructed to swallow all the capsules half way into the meal, that is, i.e., when one breakfast bun had been ingested, and one remained. No food or drink was allowed within three hours following the meals. Blinded administration was possible since the A and the B capsules were identical in appearance. Ten to 14 days after the meals were administered, blood samples were drawn and the radiation from the two Fe isotopes were determined. The total amount of blood taken from each subject for the analysis was 120 mL. Directly after drawing the blood sample a reference dose (3 mg ^59^Fe-labeled iron (II) + 30 mg of ascorbic acid dissolved in 10 mL of 0.01 M HCl) was orally administered together with 10 mL water on an empty stomach. The following morning yet another identical reference dose was served on an empty stomach. No food or drink was allowed within three hours following these reference doses. The daily mean absorption from the two reference doses was analysed in blood after a further 10–14 days (Fig 1). The total amount of blood drawn for the analysis of the reference dose absorption was 120 mL.

By relating the absorption from the capsule meals to the reference-dose-absorption, the variation depending on differences in iron absorption capacity, which primarily is dependent on iron status, was corrected. An informal agreement has been used to express absorption corresponding to a 40% reference dose absorption [41]. Thus, the iron absorption from each meal was normalized to the iron absorption that the subject would have if having a reference dose absorption of 40%. This is the percentage reference dose absorption seen in a subject with low iron stores, i.e. serum ferritin = 23 μg/l [24]. The recorded radioactivity for each subject in the different trials amounted to a total of 2 μCi from ^55^Fe and 2.0 μCi from ^59^Fe (2 x 0.5 μCi from reference-dose + 2 x 0.5 μCi from the capsule meals). The wet-chemical analysis of ^55^Fe and ^59^Fe was carried-out according to a modification of the analysis method described by Eakins and Brown [42]. Duplicates of whole-blood corresponding to 10 mg Fe were pre-treated and finally analyzed in liquid scintillator (Tri-Carb, Packard Instruments, Dallas) to determine the radiation from ^55^Fe and ^59^Fe. The absorption in percent was determined from the blood volume which was calculated from each individual’s height, weight and hemoglobin concentration [43].

## Ethics

The study was conducted according to the Ethical Principles for Medical Research Involving Human Subjects, adopted by the 18th World Medical Association General Assembly, in Helsinki, Finland, in June 1964 and amended by the 64th WMA General Assembly, in Fortaleza, Brazil, in October 2013. The study protocol was approved by the Regional Ethics Review Board in Gothenburg (Registration No. 178–13) and the Radiation Safety Committee at the Sahlgrenska University Hospital, Gothenburg, Sweden (Registration No: 13–12). The participating women gave informed written consent for participation in the study.

### Inclusion / Exclusion criteria

The subjects had to be healthy menstruating women without medication (with the exception of oral contraceptives) or any gastrointestinal, malabsorptive or metabolic diseases. The subjects were not allowed to be pregnant, lactating or to have donated blood within two months prior to the study. Neither could they take any dietary supplements (including iron) or use any probiotic containing products during the study or within two weeks prior to the study. Exclusion criteria also included infection / inflammation since an activated acute-phase reaction can cause extensive changes in body iron metabolism, including reduced iron absorption [44–46]. In the event of an activated acute-phase response, S-ferritin is falsely elevated [47]. A serum ferritin concentration of ≥120 μg/l [48,49] is above the 95th percentile of the WHO reference interval for women [50]. The reference interval is based on data from the National Health and Nutrition Examination Survey (NHANES) III-study [51], and from Custer et al [52]. A such high ferritin concentration of ≥120 μg/l is likely due to an inflammation [47]. Thus, in study 1 the exclusion criterion of S-ferritin ≥120 μg/L was chosen. However, in study 2 it was decided to be more stringent and the exclusion criteria of S-ferritin ≥60 μg/L was chosen. As an additional control of activated acute-phase response, blood was sampled for the analysis of C-reactive protein (CRP). The iron biomarkers were evaluated as a whole together with the CRP and the anamnesis in which the subjects were asked if they had experienced any signs of infection (such as fever, common cold etc.) in the previous weeks. To further increase the ability to detect any influence of activated acute-phase response during the study, blood samples destined for ferritin analysis were taken on three occasions; *1)*: At the first day of serving the meals. *2)*: Ten to fourteen days after ingesting the capsule meals and *3)*: After a further two weeks. By this it was possible to observe any discrepancy between occasions. A day-to-day variation (CV%) in S-ferritin >8% between these three occasions led to exclusion since this most likely was an expression of an activated acute-phase response [53].

### Laboratory analysis

Blood samples were collected by venous puncture. Serum iron concentration (S-Fe), total iron binding capacity (TIBC), transferrin saturation (TSAT), S-ferritin, soluble transferrin receptor (sTfR), Hb and C-reactive protein (CRP) were analyzed at an accredited reference laboratory (Clinical Chemistry Laboratory, Sahlgrenska University Hospital, Gothenburg, Sweden), according to ISO/IEC 15189 Standard for Medical Laboratories.

### Sample size

The primary hypothesis was that, in comparison with the control meal, there would be a significant increase in iron absorption after eating a meal together with freeze dried Lp299v. The sample size and power calculation was based on the fact that two different Fe isotopes were used in a sequential design, making each subject her own control, and by that, the paired student t-test can be used. When designing study 1 no previous study of this kind could be found, and by that no predictable absorption value to use in a sample size calculation existed. Consequently, Fe absorption from the control meal was predicted from the meal composition [24], and was expected to be approximately 21%. In order to, with a paired t-test and with a significance level of 0.05, have a 90% probability (i.e. a power of 90%) of observing a 10.0 ± 10.0 (SD) % difference in iron absorption [26], 13 subjects would have to be studied. Expected dropout- and exclusion frequency were estimated to be 30%, thus 18 subjects were recruited to the study.

However, although there was a significant difference in study 1 (*p* = 0.040), it was close to the margin of statistical significance. As such, there is a risk that we incorrectly reject the null hypothesis. Consequently, we decided to repeat the study and this time calculate the sample size based on the results in study 1. In order to, with a paired t-test and with a significance level of 0.05, have a 80% probability (i.e. a power of 80%) of observing a 5.0 ± 10.0 (SD) % difference in iron absorption [26], 31 subjects would have to be studied. Expected dropout- and exclusion frequency were estimated to be 15%, thus 36 subjects were recruited.

Sample size calculation was made using the StudySize 3.0 software (CREOSTAT HB, Västra Frölunda, Sweden).

## Statistics

Data were checked for normality of distribution using the Shapiro-Wilk test. Paired sample Student’s *t*-test, at a 95% confidence level, were used to analyze iron absorption differences. Student’s independent samples *t*-test, at a 95% confidence level, were used to analyze differences between study groups. The iron absorption variables were significantly skewed. Consequently, data were log-transformed before statistical analysis. For ease of interpretation, untransformed data is presented as mean and standard deviation (SD). All *p*-values were two-tailed and considered to be statistically significant if *p*<0.05. Statistical analyses were performed using IBM^®^ SPSS^®^ Statistics for Windows 22.0.0 (SPSS Inc., Chicago, IL, USA).

### Protocol deviation

Due to unexpected technical problems at the whole body counter facilities at the Sahlgrenska University hospital, the intention to utilize the whole body counter in the study had to be abandoned (S1 and S2 Files).

## Results

All absorption values are normalized so that they corresponding to a 40% reference dose absorption [41].

**Fig 2:**
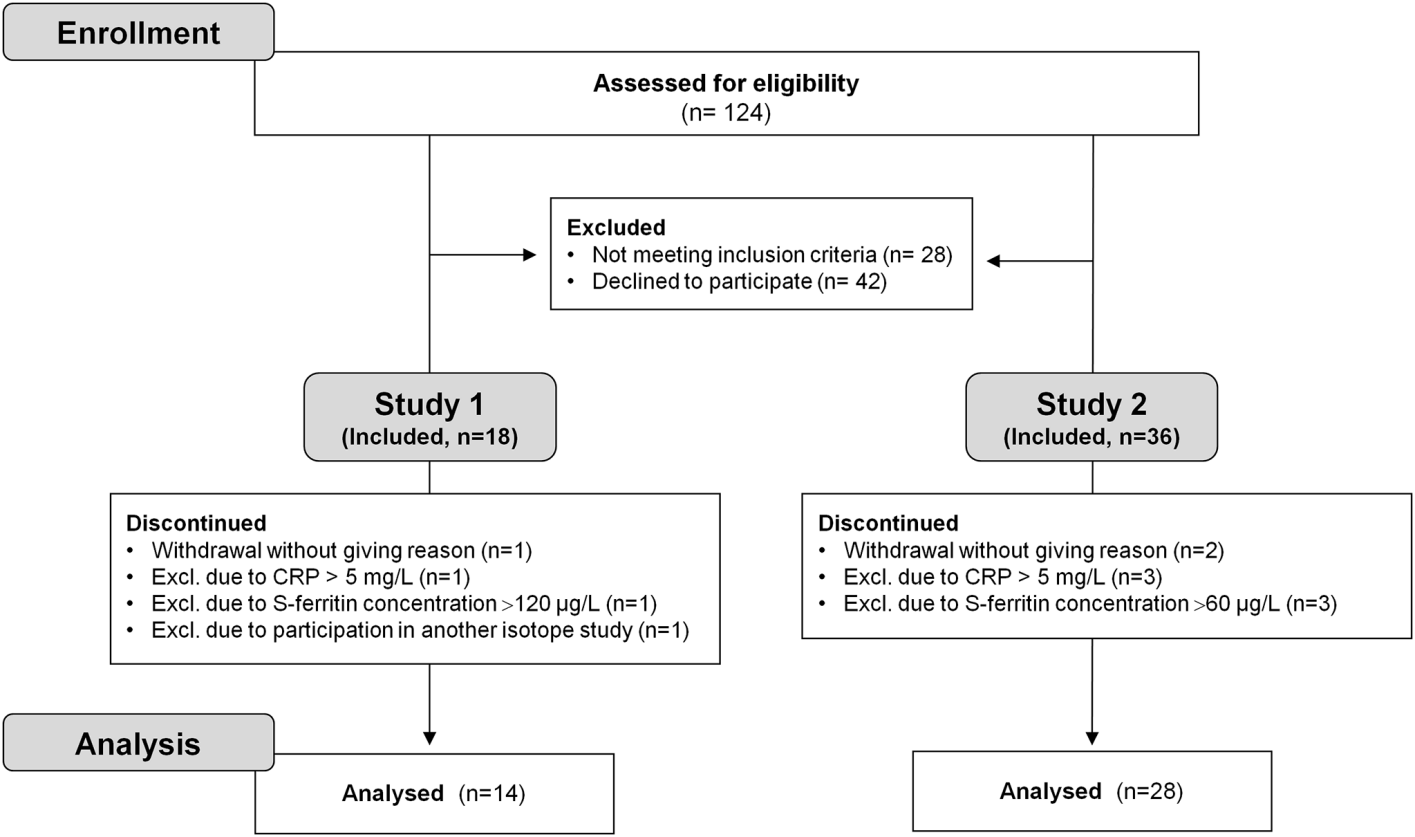
Study flowchart.

Eighteen subjects were recruited for study 1 (Fig 2). None of the participants were consuming other products containing probiotics during the four days they were served the capsule meals, or during the two weeks before the test days. One subject was excluded in connection with the first test meal as it was discovered that she recently had had another isotope test that could influence the results. Another subject decided to stop the participation at the stage of blood sampling. One subject was excluded due to a high serum ferritin concentration (120 μg/L). An additional subject was excluded due to an increased CRP (> 5 mg/L). Thus, the data analysis is based on a total of 14 females with an age between 21–40 years, an Hb-value between 123–146 g/L and a serum ferritin concentration between 8–80 μg/L (Table 1).

**Table 1:** Study subject data for study 1 and 2.

	Study 1 (n = 14)		Study 2 (n = 28)		
	mean	SD	mean	SD	*P* ^1^
**Age** (years)	26.2	4.6	25.6	6.8	*NS*
**BMI** (kg/m^2^)	22.9	3.8	22.8	2.5	*NS*
**S-ferritin** (μg/L)	30	21	27	14	*NS*
**Hemoglobin** (g/l)	135	6	134	10	*NS*
**S-Fe** (μmol/L)	15	5	16	7	*NS*
**TSAT** (%)	20	8	22	10	*NS*
**TIBC** (μmol/L)	73	10	74	12	*NS*
**CRP** (mg/L)	<5		<5		*NS*

*Abbreviations*: BMI, body mass index; CRP, C-reactive protein; S-Fe, serum iron concentration; TIBC, total iron binding capacity; TSAT, transferrin saturation; NS, Non-significant.

^***1***^ Student’s independent samples *t*-test, at a 95% confidence level, was used to analyze differences between study groups.

**Fig 3:**
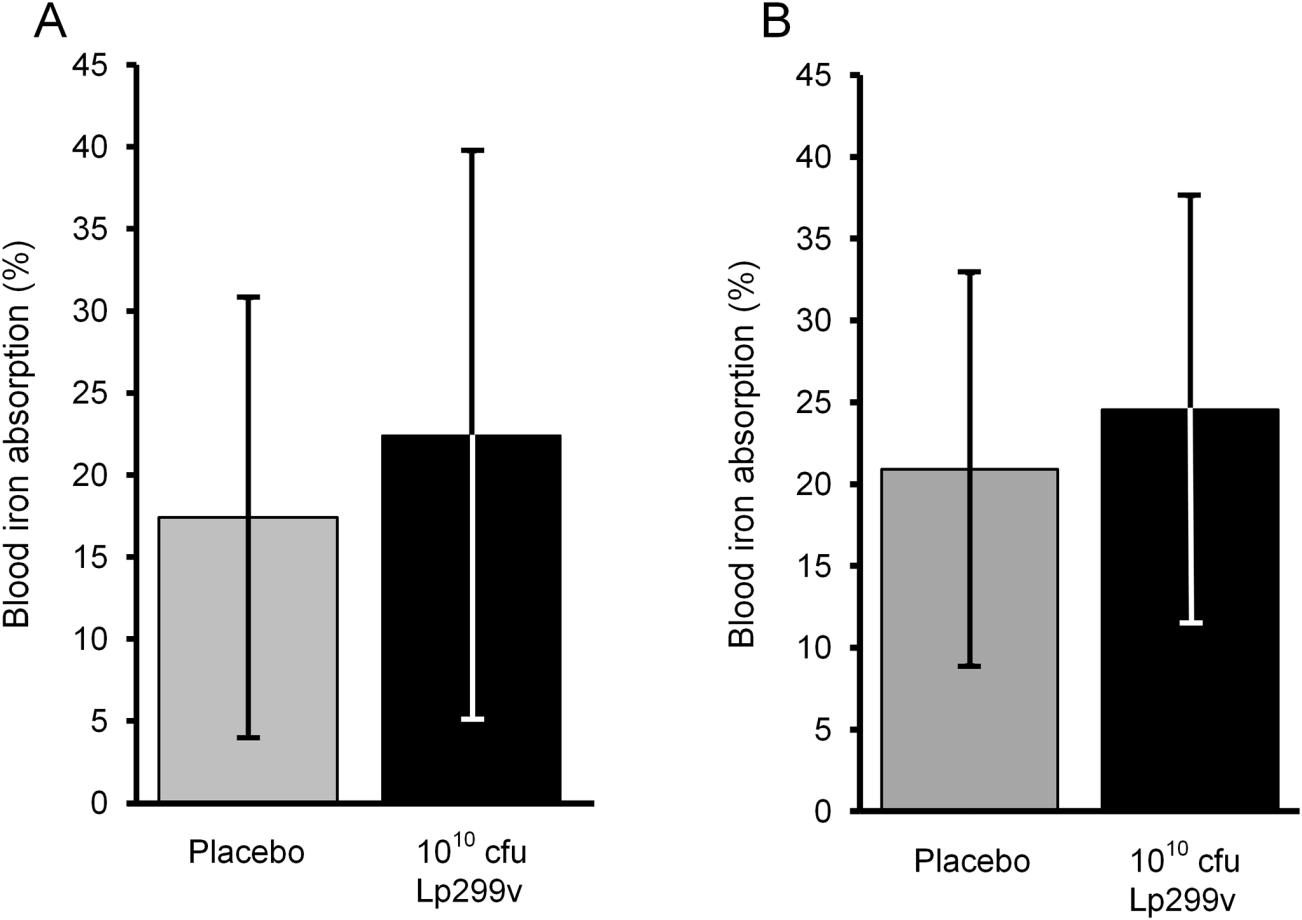
Iron absorption from a meal administered together with capsules with or without *Lactobacillus plantarum 299v* (Lp299v). Fig 3A: Study 1 (n = 14). Fig 3B: Study 2 (n = 28). The bars illustrate mean iron absorption and the whiskers cover the standard deviation (SD), and the dots illustrate individual subject data. Paired two-sample Student’s *t*-test was used to analyze differences between meals with or without Lp299v. Data were log-transformed before statistical analysis. *Abbreviations*: cfu, colony forming units.

Iron absorption from the capsule meals without Lp299v was found to be 17.4 ± 13.4% (mean ± SD). Iron absorption from capsule meals with10^10^ cfu freeze-dried Lp299v was found to be 22.4 ± 17.3% (mean ± SD). The mean difference in iron absorption between these two capsule meals was 5.0% (SD 11.0%) and statistically significant (*p* <0.040, n = 14) (Table 2, Fig 3A and S1 Data).

**Table 2:** Iron absorption from a meal administered together with capsules with or without *Lactobacillus plantarum* 299v (Lp299v).

	Study 1 (n = 14)			Study 2 (n = 28)		
	Placebo	10^10^ cfu Lp299v	*P-value*	Placebo	10^10^ cfu Lp299v	*P-value*
**Median**	**11.4**	**18.9**	*p* = 0.040	**19.6**	**25.4**	*p* = 0.003
*IQR*	*15*.*8*	*19*.*6*		*15*.*7*	*17*.*8*	
**Mean**	**17.4**	**22.4**		**20.9**	**24.5**	
*SD*	*13*.*4*	*17*.*3*		*13*.*1*	*12*.*0*	
**Log median** ^1^	**11.4**	**18.9**		**19.5**	**25.4**	
*Log IQR* ^1^	*3*.*0*	*3*.*1*		*2*.*5*	*2*.*2*	
**Log mean** ^1^	**14.0**	**18.0**		**17.0**	**21.2**	
*Log SD* ^1^	*1*.*9*	*1*.*9*		*2*.*0*	*1*.*8*	

^***1***^ Data was log-transformed, whereupon median, mean and the distribution were calculated. These values were then transformed back.

In study 2 a total of 36 subjects were recruited (Fig 2). One subject decided to drop out the day before start, and another subject dropped out after completing half the study. Three subjects were excluded due to high S-ferritin concentration (>60 μg/L), and another three subjects were excluded due to inflammation (CRP>5 mg/L). Accordingly, the data analysis of study 2 is based on a total of 28 females with an age between 19–51 years (the woman who was 51 years of age affirmed that she was menstruating), an Hb-value between 113–156 g/L and a serum ferritin concentration between 6–54 μg/L (Table 1).

In this study iron absorption from the capsule meals without Lp299v was found to be 20.9 ± 13.1% (mean ± SD). Iron absorption from the capsule meals with10^10^ cfu freeze-dried Lp299v was found to be 24.5 ± 12.0% (mean ± SD, n = 28). The mean difference in iron absorption between these two capsule meals was 3.6% (SD 8.6%) and statistically significant (*p* <0.003, n = 28) (Table 2, Fig 3B and S2 Data).

When comparing the data from both studies, there was no statistical difference between the iron absorption from the capsule meals with 10^10^ cfu Lp299v in study 1 and in study 2. Also when solely including subjects having S-ferritin <60 ug/L there was no statistical difference in iron absorption between the capsule meals with Lp299v in the two studies.

## Discussion

There seem to be a close relationship between diet, microbiota and health status [54], where dietary components are able to alter the gut microbiota [55,56]. However, survival and viability of externally added probiotic bacteria in gastric and pancreatic juice is often rather poor in *in vitro* trials [57]. Likewise, during refrigerated storage some probiotic strains exhibits loss in viability [58]. Freeze drying and microencapsulation, on the other hand, increases probiotic viability and stability [59,60], making these feasible methods for delivering the probiotic cultures in the right location of the gastrointestinal (GI) tract. Earlier studies have shown iron absorption enhancing properties of Lp299v when not lyophilized [25,26]. A previous study by Bering *et al* observed no effect on iron absorption from adding lyophilized Lp299v to oat gruel [61]. The explanation for lack of effect given in the publication was that the bacteria might not have been in a comparatively active state. However, the present study demonstrated that freeze-dried *Lactobacillus plantarum* 299v (Lp299v) is capable of increasing non-heme iron absorption from a concurrently administered meal. This is probably related to that the freeze-dried bacteria this time was included in a capsule and could be released in the stomach and thus had the possibility to reach the small intestine in an active state.

The increase in iron absorption was from a cereal meal already having a relatively high iron bioavailability since it contained a low content of phytates, the main contributor to low iron availability in cereals. Theoretically, when added to a meal with low iron bioavailability, the positive effect on iron absorption may be even more pronounced as has shown to be the case with ascorbic acid [39]. Consuming a meal of low iron bioavailability results in more iron residing in the GI lumen. By increasing the bioavailability of the iron in the diet by providing Lp299v, the dose of iron in the diet could be lower, reducing the amount of iron passing the GI tract without being absorbed. From an oxidative state of view, there are indications that redundant amounts of iron in the GI tract can result in an increased oxidative stress, with its negative aftermath [62].

By administering a fermented oatmeal drink with Lp299v to subjects for 4 weeks, Goossens *et al* significantly increased the number of lactobacilli in the fecal flora of the subjects within 1 week. However, this effect disappeared within 1 week after cessation of intake [34]. So, in order to detaining a steady colonization of Lp299v, and by that the positive effects, it seems to be necessary to keep supplying the lactobacilli.

The enhancing properties of Lp299v on iron absorption can probably be attributed to the persistency of Lp299v in the intestine. In order to reduce the day-to-day-variation in iron absorption in the present study mean absorption from two identical meals administered on two consecutive days was calculated. However, by using this methodology it is not possible to determine exactly how the Lp299v effect is mediated. Does it occur in the same way as other dietary factors influencing iron absorption, i.e., only in the common pool of the administered meal and thus having a transitory effect (i.e., same enhancing effect on day 1 as on day 2), or is it a more prolonged effect caused by intestine persistency? If the latter is the case, it could mean that the enhancing effect of administrating Lp299v differs between day 1 and day 2.

Interestingly, *in vitro* results recently showed that *Lactobacillus fermentum* has a ferric-reducing activity. This activity is proposed to be executed via an excreted molecule, p-hydroxyphenyllactic acid, which reduces ferric iron into the more bioavailable ferrous iron, and thereby boosts Fe(II) absorption through the DMT1 channels in the intestines [63]. If Lp299v also has a ferric-reducing activity is not known today.

The basis for including only women of reproductive age in the present study was the fact that this group is at higher risk of developing iron deficiency due to iron losses during menstruation. The presently used iron absorption methodology has been used extensively over the years and has been thoroughly discussed by Hallberg [36] and others. To the best of our knowledge there is nothing indicating that iron absorption would differ by mere gender. Thus, the present result is most likely applicable to other groups beside women of reproductive age.

Unfortunately, due to unexpected technical problems at the whole body counter facilities the intention to utilize the whole body counter for the present study had to be abandoned. Instead the iron absorption was solely assessed from radio iron incorporation into erythrocytes, which can be seen as a limitation of the study since it requires two important estimations. These are the percentage of absorbed iron that is incorporation in erythrocytes, and the actual blood volume of the subject. Estimates for blood volume are usually calculated from sex, weight, and height. In healthy subjects having normal iron status, approximately 80% of absorbed iron will be incorporated into erythrocytes. However, this figure can differ depending on e.g. iron status, or presence of inflammation or infection. Nevertheless, since each subject is her own control, this limitation has most likely only a potential effect on the accuracy, not the iron absorption ratio between meal A and meal B, which is the primary outcome of the present study.

*In conclusion*, the results brought forward in the present study further strengthen the evidence that Lp299v enhances iron absorption when concurrently added to a meal. Thus, Lp299v provide a valuable alternative for increasing iron bioavailability in the diet of vulnerable individuals who are in need of increasing their iron uptake due to high requirements. By increasing the bioavailability of iron in the diet by providing Lp299v it could be possible to lower the amount of iron added to a meal, and therefore reduce the amount of iron residing in the GI tract leading to negative health consequences.

## Supporting information

S1 File Bilaga 2- STUDY PROTOCOL. (DOC)

S2 File Bilaga 2- STUDY PROTOCOL ENG. (DOC)

S3 File Trendstatement_TREND_Checklist. (PDF)

S1 Data Data—Study 1. (PDF)

S2 Data Data—Study 2. (PDF)
