# Supplementary material for: Freeze-dried Lactobacillus plantarum 299v increases iron absorption in young females—Double isotope sequential single-blind studies in menstruating women
Source: PLoS One. 2017 Dec 13;12(12):e0189141. doi: 10.1371/journal.pone.0189141 (PMC5728536; doi:10.1371/journal.pone.0189141)
Supplement: S1 File — (DOC) [file pone.0189141.s001.doc]

# *Studieprotokoll*

***Bilaga nr 2***

**Studie av järnabsorption från kapslar**

**innehållande lactobaciller och järn**

**Projektledare:** Lena Hulthén

Avd för Invärtesmedicin och Klinisk Näringslära

Sahlgrenska Akademin vid Göteborgs Universitet

SE-405 30 Göteborg

Tel.: 031-786 37 14

E-post: Lena.Hulthen@medfak.gu.se

**Syfte:**

Målet med föreliggande projekt är att studera järnabsorption från en frystorkad probiotika-innehållande (*Lactobacillus plantarum* 299v) kapsel med förmåga att optimera absorptionen av järn.

**OMRÅDESÖVERSIKT:**

Järnbrist och låg järnstatus är vanligt förekommande hos barn, ungdomar och kvinnor i barnafödande ålder i både västvärlden och i utvecklingsländer. Detta kan leda till järnbristanemi, minskad arbetskapacitet och ett sämre immunförsvar (*Dallman et al. 1980; Milman 1996; WHO 2012*). Järnbrist finns hos 30% av fertila kvinnor i Sverige (*Abrahamsson et al. 2006*) och i Europa har 8-33% av de fertila kvinnorna tomma järnlager (*Hercberg et al. 2001*). Detta beror på att menstruerande kvinnor har höga järnbehov och inte tillräckligt högt intag av järn och/eller för låg biotillgänglighet av järnet i kosten. Att inta livsmedel som är rika på järn och innehåller ämnen som ökar järnabsorptionen är därför viktigt.

Den 1 januari 1995 togs järnberikningen av mjöl bort i Sverige, en berikning som då pågått drygt 50 år med målet att minska risken för att utveckla järnbrist. För närvarande finns det alltså ingen allmän berikning av livsmedel med järn i Sverige och personer med järnbrist hänvisas till att välja livsmedel rika på järn, livsmedel som ökar biotillgängligheten av järn och kosttillskott med järn för att förbättra järnstatusen. I länder som fortfarande berikar livsmedel med järn har det visats att det är svårt att få en förbättrad järnstatus om kosten har låg tillgänglighet på järn (*Hoppe et al. 2008*). Slutsatsen är att biotillgängligheten av järnet i kosten är av stor betydelse för järnstatus.

Kostens järn förekommer i två former, antingen som hemjärn som återfinns i kött och köttprodukter eller som icke-hemjärn i spannmål, grönsaker, baljväxter, bönor, frukt etc. (*Abrahamsson et al. 2006*). Icke-hemjärnet dominerar i kosten och omfattar cirka 90-95% av det totala intaget av järn. Absorptionen av båda järnformerna sker främst i den övre delen av tunntarmen. Hemjärn är den mest biotillgängliga formen av järn varav 20-30% kan absorberas. Majoriteten av kostens icke-hemjärn återfinns som järn (III) (FeIII) och måste konverteras till tvåvärt järn (FeII) för att kunna absorberas. Detta leder till en låg biotillgänglighet och endast 1-10% absorberas. Absorptionen av icke-hemjärn i tarmen beror också på kroppens järnstatus och på kostens sammansättning. En rad kostfaktorer påverkar upptaget av icke-hemjärn. Intag av askorbinsyra och kött stimulerar upptaget medan kalcium, polyfenoler (t ex i te, kaffe, grönsaker) och fytater (t ex i fullkornscerealier) hämmar upptaget. En annan faktor som kan öka absorptionen av järn är probiotika (*Bering et al. 2006*).

Enligt WHO definieras probiotika som ”levande mikroorganismer som när de intas i tillräcklig mängd medför hälsovinster för konsumenten”. Ett flertal olika probiotiska stammar har visats kunna påverka kroppens immunförsvar och återställa mag-tarmhälsan (*Johansson 1993; Nobaek 2000; Asp 2004; Boirivant 2007*). Bland de mest studerade finns *Lactobacillus plantarum* 299v, vilka visats öka järnabsorptionen (*Bering et al. 2006; Hulthén och Hoppe, 2007*). Målet med föreliggande projekt är att studera en ny frystorkad probiotika-baserad produkt med förmåga att optimera absorptionen av järn. En frystorkad formulering är optimalt eftersom produkten då dels kan tillsättas till livsmedel och även nyttjas som ett separat kosttillskott.

**Försökspersoner:**

Totalt skall 5 stycken serier i grupper om 20 forskningspersoner (tot. n=100 st) utföras.

**Inklusionskriterier:**

De personer som skall ingå är frivilliga, friska kvinnor med Hb>117 g/L och serumferritin 15-55 µg/L.

**Exklusionskriterier:**

- Gastrointestinala sjukdomar
- Metabola sjukdomar
- Gravid eller ammande
- Medicinering (undantaget orala preventivmedel)
- Intag av kosttillskott (inkl. järn) under studien samt närmare än två veckor innan studien.
- Blodgivning närmare än två månader innan studien.

**Metod/Design:**

Järnupptaget kommer att studeras i fem grupper om 20 forskningspersoner där varje individ serveras antingen en kapsel innehållande 4,2 mg järn (A), eller en kapsel innehållande 4,2 mg järn + lactobaciller Lp299v (B). I varje grupp ges kapslar med eller utan lactobaciller alternerande under fyra påföljande dagar med ordningsföljden AABB. För att kunna avgöra absorberad mängd järn märks järnet i kapslarna (A och B) med ^55^Fe, respektive ^59^Fe.

- **Kapsel A** innehåller 4,2 mg järn men inga lactobaciller.
- **Kapsel B** innehåller 4,2 mg järn + olika koncentrationer Lp299v (6, 10, 20, 40, eller 80 Gigacfu beroende på försök).

**Absorptionsbestämning:**

Metodiken som skall användas är den s.k. ”dubbel-isotoptekniken” (eng. The Extrinsic Tag Technique). Dubbelisotoptekniken har använts vid Sahlgrenska Universitetssjukhuset för humanstudier i över 30 år och är väl validerad. Tekniken är den enda i Europa och därmed unik. En av fördelarna med att använda två olika isotoper, är att varje person blir sin egen kontroll. Tio till 16 dagar efter intaget av kapslarna utförs en helkroppsmätning på varje försöksperson för att bestämma strålningen från ^59^Fe. Helkroppsmätningen av ^59^Fe äger rum vid Radiofysiska institutionen, Sahlgrenska Universitetssjukhuset. Med hjälp av helkropps-räknaren kan den totala mängden av absorberad gammastrålning bestämmas m.h.a. en standard och en korrektionsfaktor baserad på kroppsvikt och längd. Strålningen från ^55^Fe (β-strålning) kan ej detekteras med helkroppsmätaren. Därför tas samma dag även ett blodprov vari strålningen från de båda Fe-isotoperna bestäms. Mängden blod som tas av varje försöks-person uppgår till ca. 1/3 av en vanlig blodgivning. Genom helkroppsmätningen kan totala kroppsretentionen av ^59^Fe bestämmas. Tillsammans med analysvärdet från blodprovet, som ger den relativa absorptionen av de båda isotoperna, möjliggör detta beräkning av total retention (d.v.s. båda isotoperna) samt total absorption från kapslarna. Efter helkropps-mätningen och blodprovstagningen administreras en referensdos (10 ml 0.01 mol HCl innehållande 3 mg ^59^Fe-märkt järn(II) + 30 mg askorbinsyra) peroralt på fastande mage. Till denna referensdos, serveras 100 ml vatten. Morgonen efter ges återigen en referensdos på fastande mage. Ingen mat eller dryck tillåts inom tre timmar efter intag av dessa referens-doser. Absorptionen från referensdosen mäts efter ytterligare två veckor i helkroppsräknaren. Med hjälp av denna mätning kalibreras övriga mätningar för individuella olikheter i absorption av järn. Genom att relatera absorptionen från kapslarna till denna referensdos-absorption kan variationen i absorption som beror på olikheter i Fe-status korrigeras.

**TOTALT Ingiven radioaktivitet**

Den ingivna radioaktiviteten för varje forskningsperson i de olika försöken uppgår totalt till 2.0 µCi (0,074 MBq) från ^55^Fe samt 3.0 µCi (0,111 MBq) från ^59^Fe (2 x 0.75 µCi från referensdosen + 2 x 0.75 µCi från kapslarna). Den våtkemiska analysen utförs enligt en modifiering av analysmetoden beskriven av Eakins och Brown. Dubbletter av helblod motsvarande 10 mg Fe upparbetas och analyseras slutligen i vätskescintillator (Tri-Carb, Packard Instruments, Dallas) för att bestämma strålningen från ^55^Fe och ^59^Fe.

**Inmärkning med radioisotoper**

Varje kapsel kommer att märkas med radioisotop (som FeCl_3_) strax före servering.

**Rekrytering av fORSKNINGSpersoner**

Forskningspersonerna kommer att utgöras av frivilliga studenter vid Sahlgrenska Akademin vid Göteborg Universitet och Chalmers tekniska högskola, vilka uppfyller studiens inklusion-kriterier samt är villiga till att medverka. Ersättning för deltagande i försöken utgår med 1000 kr efter fullt genomförd studie.

**Antal försökspersoner**

Vi avser att i dessa försök använda oss av den s.k. "dubbelisotoptekniken". En av fördelarna med denna teknik är att varje forskningsperson blir sin egen kontroll. Varje försöksperson serveras varje kapsel två dagar för att reducera inflytande av dag-till-dag-variation. Den primära hypotesen är att järnabsorptionen från kapslar innehållande 4,2 mg järn tillsammans med Lactobacillus plantarum 299v kommer att vara signifikant högre jämfört med placebo-kapslar (4,2 mg Fe, men EJ Lactobacillus plantarum 299v). För att med en signifikansnivå av 0,05 ha en 90 %-ig möjlighet med ett parat t-test kunna observera en skillnad i järnabsorption på 10 %-enheter samt en standardavvikelse på 9 %-enheter från de kapslar med och utan Lactobacillus plantarum 299v krävs 18 försökspersoner. Med en förväntad avhoppsfrekvens om <20% innebär det att ca 20 forskningspersoner bör ingå i respektive försöksgrupp vid studiens start. Totalt 100 forskningspersoner/5 försöksgrupper.

**Analysvariabler:**

Blodprover destinerade för analys av serumferritin (SF), serumjärn (S-Fe), transferrinmättnad (TSAT), total järnbindningskapacitet (TIBC), hemoglobin (Hb) kommer att insamlas. För att undvika systematiska fel introducerade av infektion kommer vid dessa tidpunkter även blod-prov att analyseras med avseende på C-reaktivt protein (CRP) och sänkningreaktion (SR). Försökspersonerna kommer även muntligen att tillfrågas om de haft indikationer på infektion under de föregående veckorna.

**Statistisk metod:**

Medelvärde, standardavvikelse, geometriskt medelvärde och 95%-igt konfidensintervall kommer att användas för att beskriva materialet. Medelvärden kommer att jämföras med hjälp av tvåsidigt t-test, vid en konfidensnivå av 0,05.

**Etiska överväganden**

Forskningspersonerna kommer att informeras både skriftligt och muntligt om syftet och tillvägagångssättet i studien. Samtycke till deltagande inhämtas genom underskrift av informationsbrevet. Detta är dock ej bindande, vilket innebar att försökspersonen vid vilken tidpunkt som helst kan lämna studien utan angivande av orsak. Godkännande kommer att sökas vid regionala etikprövningsnämnden samt vid strålskyddskommittén vid Sahlgrenska Universitetssjukhuset.

**Koordinering**

Projektledaren Lena Hulthén är ansvarig för koordineringen av studien.

**Monitorering**

Elisabeth Gramatkovski samt Michael Hoppe vid avd. Invärtesmedicin och Klinisk närings-lära är ansvariga för genomförandet av studien.

**Behandling av data och statistik**

Projektledaren Lena Hulthén, samt Michael Hoppe och Elisabeth Gramatkovski vid avd. för Invärtesmedicin och Klinisk näringslära är ansvariga för behandling av data och statistik.

**Tidsschema**

Studien avses starta April-Juni 2013.

**REFERENSER:**

Abrahamsson L, Andersson A, Becker W, Nilsson G. (2006). Näringslära för högskolan. Liber.

Asp NG., Möllby R., Norin L. and Wadström T.(2004) Probiotics in gastric and intestinal disorders as functional food and medicine. Scandinavian Journal of Nutrition 48: 15-25.

Bering, S., Suchdev, S., Sjøltov, L., Berggren, A., Tetens, I., Bukhave, K. (2006). A lactic acid-fermented oat-gruel increases non-haem absorption from a phytate-rich meal in healthy women of childbearing age. British J Nutr 96:1-6.

Boirivant M. and Strober W. (2007) The mechanism of action of probiotics. Curr Opin Gastroenterol 23: 679-692

Dallman, P.R., Siimes, M.A., Stekel, A. (1980). Iron deficiency in infancy and childhood. Am J Clin Nutr 33:86-118.

Hertcberg, S., Preziosi, P., Galan, P. (2001). Iron deficiency in Europe. Public Health Nutrition 4: 537-545.

Hoppe M, Hulthén L, Hallberg L. (2008). The importance of bioavailability of dietary iron in relation to the expected effect from iron fortification. Eur J Clin Nutr. 62:761-9.

Hulthén, L., Hoppe, M. (2007). Iron absorption in an iron supplemented fruit drink containing lactobacilli. Report. Göteborg University, Sweden.

Johansson M.-L., Molin G., Jeppsson B., Nobaek S., Ahrné, S. and Bengmark, S. (1993) Administration of different *Lactobacillus* strains in fermented oatmeal soup: *in vivo* colonization of human intestinal mucosa and effect on the indigenous flora. Applied and Environmental Microbiology 59: 15-20.

Milman, N. (1996). Serum ferritin in Danes: studies of iron status from infancy to old age, during blood donation and pregnancy. Int J Hematol 63:103-135.

Nobaek S., Johansson M.-L., Molin G., Ahrné S. and Jeppsson B. (2000) Alteration of intestinal microflora is associated with reduction in abdominal blaoting and pain in patients with irritable bowel syndrome (IBS). American Journal of Gastroenterology 95: 1231-1238.

World Health Organisation. (2012). Homepage: http://www.who.int/nutrition/topics/ida/en/
