# Supplementary material for: Freeze-dried Lactobacillus plantarum 299v increases iron absorption in young females—Double isotope sequential single-blind studies in menstruating women
Source: PLoS One. 2017 Dec 13;12(12):e0189141. doi: 10.1371/journal.pone.0189141 (PMC5728536; doi:10.1371/journal.pone.0189141)
Supplement: S2 File — (DOC) [file pone.0189141.s002.doc]

# *Study protocol*

**Study of iron absorption from capsules**

**containing lactobacilli and iron**

**PROJECT MANAGER:** Lena Hulthén

Department of Internal Medicine and Clinical Nutrition

Sahlgrenska Academy at the University of Gothenburg

SE-405 30 Gothenburg

      Tel .: 031-786 37 14

       E-mail: Lena.Hulthen@medfak.gu.se

**PURPOSE**

The aim of the present project is to study iron absorption from a freeze dried probiotics (Lactobacillus plantarum 299v) containing capsule.

**RESEARCH OVERVIEW**

Iron deficiency and low iron status are common in children, adolescents and women of childbearing age in both the western world and developing countries. This can lead to iron deficiency anemia, reduced labor capacity and a impaired immune system (*Dallman et al., 1980; Milman 1996; WHO 2012*). Iron deficiency is found in 30% of fertile women in Sweden (*Abrahamsson et al. 2006*). In Europe, 8-33% of the fertile women have empty iron stores (*Hercberg et al., 2001*). This is due to the fact that menstruating women have high iron needs and not enough iron and / or low iron bioavailability in their diets. Eating a diet rich in iron and iron absorption enhancing dietary factors are therefore important.

On January 1, 1995, iron enrichment of flour was removed in Sweden, an enrichment that lasted more than 50 years with the aim of reducing the risk of iron deficiency. At present, there is no general iron enrichment of food in Sweden, and people with iron deficiency are referring to choosing iron rich food, and iron supplements to improve iron status. In countries still enriching food with iron, it has been shown that it is difficult to get an improved iron status if the diet has low iron availability (*Hoppe et al., 2008*). The conclusion is that the bioavailability of the iron in the diet is of major importance for iron status.

The iron of iron is found in two forms, either as heme-iron found in meat and meat products or as non-heme-iron in cereals, vegetables, legumes, beans, fruit, etc. (*Abrahamsson et al., 2006*). Non-heme-iron is the predominating form of iron in the diet and comprise about 90-95% of the total iron intake. The absorption of both iron forms occurs mainly in the upper part of the small intestine. Heme-iron is the most bioavailable form of iron, of which 20-30% can be absorbed. The majority of the diet's non-heme-iron is found as iron (III) (FeIII) and must be converted to divalent iron (FeII) in order to be absorbed. This leads to low bioavailability and only 1-10% is absorbed. The absorption of non-heme-iron in the intestine also depends on iron status and on the composition of the diet. A number of dietary factors affect the uptake of non-heme-iron. Intake of ascorbic acid and meat stimulates the absorption while calcium, polyphenols (for example, in tea, coffee, vegetables) and phytates (eg in whole grain cereals) inhibit the absorption. Another factor capable of increasing the absorption of iron is probiotics (*Bering et al., 2006*).

According to the WHO, probiotics are defined as "living microorganisms, which when consumed in a sufficient amount bring health benefits to the consumer." A number of different probiotic strains have been shown to affect the body's immune system and restore gastrointestinal health (*Johansson 1993; Nobaek 2000; Asp 2004; Boirivant 2007*). Among the most studied are Lactobacillus plantarum 299v, which has been shown to increase iron absorption (*Bering et al. 2006; Hulthén and Hoppe, 2007*). The aim of the present project is to study a freeze dried probiotic based product capable of optimizing the absorption of iron. A freeze dried formulation is optimal because it can either be added to food, or used as a dietary supplement.

**SUBJECTS**

In total, 5 trials with 20 subjects in each (tot. n = 100) will be performed.

**INCLUSION CRITERIA**

Subjects to be included are voluntary, healthy women with Hb 117 g / L and serum ferritin 15-55 μg / L.

**EXCLUSION CRITERIA**

- Gastrointestinal disorders
- Metabolic diseases
- Pregnant or breast-feeding
- Medication (except oral contraception)
- Intake of dietary supplements (including iron) during the study and closer to two weeks before the start of the study.
- Blood donation more than two months before the study.

**METHODOLOGY / DESIGN**

Iron absorption will be studied in five groups of 20 subjects in each. In each group, capsules containing 4.2 mg iron (A), or capsules containing 4.2 mg iron + lactobacilli Lp299v (B) are given at four consecutive days to each subject in the order AABB. To determine the amount of iron absorbed, the iron in the capsules (A and B) is labeled with ^55^Fe and ^59^Fe respectively.

- **Capsule A** contains 4.2 mg iron but no lactobacilli.
- **Capsule B** contains 4.2 mg iron + different concentrations Lp299v (6, 10, 20, 40, or 80 Gigacfu depending on trial).

The methodology to be used is the so-called Extrinsic Tag Technique. This methodology has been used at Sahlgrenska University Hospital for Human Studies for over 30 years and is well validated. One of the advantages of using two different isotopes is that each person becomes his own control. Ten to 16 days after the intake of the capsules, a whole body counting is performed on each subject to determine the radiation from ^59^Fe. The full body measurement of ^59^Fe takes place at the Radio-Physical Department, Sahlgrenska University Hospital. With the aid of the whole body counter, the total amount of absorbed gamma radiation can be determined, by using a standard and a correction factor based on body weight and height. The radiation from ^55^Fe (β-radiation) cannot be detected by the whole body counter. Thus, on the same day, a blood sample is also taken in which the radiation from the two Fe isotopes is determined. The amount of blood drawn from each subject is approximately 1/3 of a ordinary blood donation. By using the whole body counter, the total body retention of 59Fe can be determined. Together with the retention result from the blood sample, which provides the relative absorption of the two isotopes, this allows for total retention (i.e., both isotopes) and total absorption from both capsules. After the whole body counting and the blood sampling, a reference dose (10 ml 0.01 mol HCl containing 3 mg ^59^Fe-labeled iron (II) + 30 mg ascorbic acid) is administered orally on an empty stomach. Together with the reference dose, an additional 100 ml of water is administered. The following morning, yet another reference dose is administered on fasting stomach. No food or drink is allowed within three hours after drinking these reference doses. The iron absorption from the reference dose is measured after a further two weeks in the whole body counter. By using this reference dose absorption, other measurements are calibrated for individual differences in iron absorption. By relating the iron absorption from the capsules to this reference dose absorption, individual variations in absorption due to differences in Fe status can be corrected.

**RADIOACTIVITY**

The reported radioactivity for each subject in the various trials amounts to 2.0 μCi (0.074 MBq) from ^55^Fe and 3.0 μCi (0.111 MBq) from ^59^Fe (2 x 0.75 μCi from the reference dose + 2 x 0.75 μCi from the capsules). The wet chemical analysis is performed according to a modification of the assay method described by Eakins and Brown. Duplicates of blood corresponding to 10 mg Fe is finally processed and analyzed in liquid scintillator (Tri-Carb, Packard Instruments, Dallas) to determine the radiation from ^55^Fe and ^59^Fe.

**LABELLING WITH RADIO ISOTOPES**

Each capsule will be labeled with radioisotope (FeCl_3_) just prior to serving.

**RECRUITMENT OF SUBJECTS**

The subjects will be volunteering students at the Sahlgrenska Academy at the University of Gothenburg, and Chalmers University of Technology, who meet the study's inclusion criteria and are willing to participate. Compensation for participation in the trial is SEK 1,000.

**NUMBER OF SUBJECTS**

We intend to use the Extrinsic Tag Technique. One of the benefits of this technique is that each subject becomes her own control. Each subject is served each capsule two days in order to reduce the influence of day-to-day variation in iron absorption. The primary hypothesis is that the iron absorption from capsules containing 4.2 mg iron together with Lactobacillus plantarum 299v will be significantly higher compared to placebo capsules (4.2 mg Fe without Lactobacillus plantarum 299v). In order to be able to observe a difference in iron absorption of 10% and a standard deviation of 9% from the capsules (with and without Lactobacillus plantarum 299v) 18 subjects in each of the two groups are needed (at a significance level of 0.05, and a 90% possibility). With an expected drop-out rate of <20%, it means that approximately 20 subjects should be included in each group. A total of 100 subjects / 5 trials.

**ANALYSIS VARIABLES**

Blood samples destined for the analysis of serum ferritin (SF), serum iron (S-Fe), transferrin saturation (TSAT), total iron binding capacity (TIBC), hemoglobin (Hb) will be collected. In order to minimize systematic errors introduced by infection, blood samples will also be analyzed for C-reactive protein (CRP) and erythrocyte sedimentation rate (ESR). The subjects will also be asked orally if they have had indications of infection the previous weeks.

**STATISTICAL METHOD**

Mean, standard deviation, geometric mean and 95% confidence interval will be used to describe the material. Mean values ​​will be compared using two-sided t-test, at a confidence level of 0.05.

**ETHICAL CONSIDERATIONS**

The subjects will be informed both written and orally about the purpose and means of conducting the study. A consent to participate is to be signed by the subject before the study started, but the subject could leave the study at any time without giving any explanation. Approval will be applied from the Regional Ethics Committee in Gothenburg, as well as the Radiation Protection Committee and Radiation Ethics at the Sahlgrenska University Hospital.

**COORDINATION**

Project leader is Professor Lena Hulthén.

**MONITORING**

Elisabeth Gramatkovski and Michael Hoppe at the dept. of Internal Medicine and Clinical Nutrition are responsible for the implementation of the study.

**TREATMENT OF DATA AND STATISTICS**

Project leader Lena Hulthén, as well as Michael Hoppe and Elisabeth Gramatkovski at the dept. of Internal Medicine and Clinical Nutrition are responsible for processing data and statistics.

**TIME SCHEDULE**

The study is intended to start April-June 2013.

**REFERENCES**

Abrahamsson L, Andersson A, Becker W, Nilsson G. (2006). Näringslära för högskolan. Liber.

Asp NG., Möllby R., Norin L. and Wadström T.(2004) Probiotics in gastric and intestinal disorders as functional food and medicine. Scandinavian Journal of Nutrition 48: 15-25.

Bering, S., Suchdev, S., Sjøltov, L., Berggren, A., Tetens, I., Bukhave, K. (2006). A lactic acid-fermented oat-gruel increases non-haem absorption from a phytate-rich meal in healthy women of childbearing age. British J Nutr 96:1-6.

Boirivant M. and Strober W. (2007) The mechanism of action of probiotics. Curr Opin Gastroenterol 23: 679-692

Dallman, P.R., Siimes, M.A., Stekel, A. (1980). Iron deficiency in infancy and childhood. Am J Clin Nutr 33:86-118.

Hertcberg, S., Preziosi, P., Galan, P. (2001). Iron deficiency in Europe. Public Health Nutrition 4: 537-545.

Hoppe M, Hulthén L, Hallberg L. (2008). The importance of bioavailability of dietary iron in relation to the expected effect from iron fortification. Eur J Clin Nutr. 62:761-9.

Hulthén, L., Hoppe, M. (2007). Iron absorption in an iron supplemented fruit drink containing lactobacilli. Report. Göteborg University, Sweden.

Johansson M.-L., Molin G., Jeppsson B., Nobaek S., Ahrné, S. and Bengmark, S. (1993) Administration of different *Lactobacillus* strains in fermented oatmeal soup: *in vivo* colonization of human intestinal mucosa and effect on the indigenous flora. Applied and Environmental Microbiology 59: 15-20.

Milman, N. (1996). Serum ferritin in Danes: studies of iron status from infancy to old age, during blood donation and pregnancy. Int J Hematol 63:103-135.

Nobaek S., Johansson M.-L., Molin G., Ahrné S. and Jeppsson B. (2000) Alteration of intestinal microflora is associated with reduction in abdominal blaoting and pain in patients with irritable bowel syndrome (IBS). American Journal of Gastroenterology 95: 1231-1238.

World Health Organisation. (2012). Homepage: http://www.who.int/nutrition/topics/ida/en/
