# Supplementary material for: Freeze-dried Lactobacillus plantarum 299v increases iron absorption in young females—Double isotope sequential single-blind studies in menstruating women
Source: PLoS One. 2017 Dec 13;12(12):e0189141. doi: 10.1371/journal.pone.0189141 (PMC5728536; doi:10.1371/journal.pone.0189141)
Supplement: S1 Data — (PDF) [file pone.0189141.s004.pdf]

										Fe absorption			Absorption adjusted to reference dose abs of 40%	
										With LP299v	*Without LP299v*			
**ID**	**BMI**	**Age** (y)	**Ferritin**	**S-Fe**	**TIBC**	**TSAT**	**TfR**	**Hb**	**CRP**	**59-Fe** *(blood)*	**55-Fe** *(blood)*	**Reference dose**	*With LP299v*	*Without LP299v*
1	20,4	23,8	8	5	84	6%	5,3	138	1	59,1	34,2	32,7	72,3	41,8
2	29,5	39,6	11	9	78	11%	4,3	136	1	51,5	37,8	83,4	24,7	18,1
3	18,8	31,4	32	19	68	28%	3,5	139	1	13,3	13,4	53,4	10,0	10,0
4	21,5	27,9	29	20	67	30%	4,6	142	0	29,2	10,8	58,0	20,1	7,4
5	27,8	23,6	68	18	72	25%	2,4	146	1	7,3	6,7	34,5	8,5	7,8
6	21,3	24,5	80	13	70	19%	3,3	134	1	2,7	2,5	12,4	8,7	8,1
7	28,1	26,5	20	9	62	15%	3,1	135	1	40,0	43,0	67,7	23,6	25,4
8	23,5	24,9	23	19	59	33%	3,4	126	1	10,7	10,7	37,9	11,3	11,3
9	25,0	26,6	20	19	66	29%	3,2	135	1	30,3	41,5	33,4	36,3	49,7
10	21,8	21,4	23	10	67	15%	2,4	123	1	9,1	6,6	20,5	17,8	12,9
11	18,5	24,2	35	19	85	22%	3,1	137	1	24,3	21,0	36,1	26,9	23,3
12	16,4	23,2	8,5	14	93	15%	4,4	131	1	34,7	11,2	39,2	35,4	11,4
13	24,3	23,6	32	19	69	27%	2,6	129	1	8,6	9,7	36,0	9,6	10,8
14	23,2	24,9	26	10	87	11%	2,3	138	1	8,5	5,5	38,1	8,9	5,8
**Mean**	**22,9**	**26,2**	**30**	**15**	**73**	**20%**	**3,4**	**135**	**1**	**23,5**	**18,2**	**41,7**	**22,4**	**17,4**
**SD**	**3,8**	**4,6**	**21**	**5**	**10**	**8%**	**0,9**	**6**	**0**	**17,9**	**14,5**	**18,4**	**17,3**	**13,4**
Median	***22,5***	***24,7***	***25***	***16***	***70***	***21%***	***3,3***	***136***	***1***	***18,8***	***11,0***	***37,0***	***18,9***	***11,4***
