# Supplementary material for: Freeze-dried Lactobacillus plantarum 299v increases iron absorption in young females—Double isotope sequential single-blind studies in menstruating women
Source: PLoS One. 2017 Dec 13;12(12):e0189141. doi: 10.1371/journal.pone.0189141 (PMC5728536; doi:10.1371/journal.pone.0189141)
Supplement: S2 Data — (PDF) [file pone.0189141.s005.pdf]

										Fe absorption			Absorption adjusted to reference dose abs of 40%	
										With LP299v	*Without LP299v*			
**ID**	**BMI**	**Age** (y)	**Ferritin**	**S-Fe**	**TIBC**	**TSAT**	**TfR**	**Hb**	**CRP**	**59-Fe** *(blood)*	**55-Fe** *(blood)*	**Reference dose**	*With LP299v*	*Without LP299v*
1	25,9	22,7	21	12	76	15%	3,4	123	1	68,7	36,5	74,8	36,7	19,5
2	19,6	23,6	13	15	77	19%	3,2	144	1	32,8	33,9	68,7	19,1	19,7
3	26,2	23,5	15	15	77	19%	3,4	132	1	33,8	39,9	49,3	27,4	32,4
4	21,9	21,8	12	16	90	18%	3,8	118	1	15,1	24,5	22,4	27,0	43,8
5	23,8	24,3	15	12	79	15%	3,5	134	2	37,8	37,9	63,4	23,8	23,9
6	23,6	23,9	21	33	100	33%	3,6	135	4	48,4	29,2	53,7	36,1	21,8
7	27,1	22,2	30	12	76	15%	3,3	145	3	43,5	49,8	36,3	47,9	54,9
8	22,8	32,2	24	19	80	23%	2,2	136	2	15,5	18,4	14,9	41,6	49,4
9	25,8	21,8	12	7	82	8%	3,2	130	1	60,4	29,5	77,5	31,2	15,2
10	21,0	22,7	34	23	82	28%	2,5	131	1	14,9	13,2	31,2	19,1	16,9
11	22,4	22,3	26	21	71	30%	5,0	131	1	54,5	37,6	46,5	46,9	32,3
12	19,4	25,8	18	11	87	13%	4,0	142	3	18,5	21,6	27,2	27,2	31,8
13	22,2	24,9	31	24	65	37%	2	130	1	6,8	4,6	48,8	5,6	3,8
14	20,1	22,4	38	13	75	18%	3,9	150	1	12,5	5,9	40,0	12,5	5,9
15	23,2	25,1	15	9	64	13%	2,9	133	1	30,4	12,0	60,5	20,1	7,9
16	20,5	51,2	54	16	57	29%	2,6	132	1	6,1	4,6	29,5	8,3	6,2
17	21,6	31,7	44	13	52	25%	2,0	135	1	18,8	17,6	49,5	15,2	14,2
18	28,2	23,2	10	5	100	5%	5,4	131	1	44,0	35,2	53,3	33,0	26,4

19	25,5	29,2	19	14	86	17%	3,3	134	1	59,9	32,9	63,8	37,6	20,6
20	22,7	29,3	32	37	72	51%	2,6	142	1	13,5	9,5	35,7	15,1	10,6
21	23,9	27,6	34	16	64	25%	4,6	156	1	23,3	35,3	57,4	16,2	24,6
22	21,8	39,5	14	20	67	30%	2,3	124	1	45,0	38,9	59,2	30,4	26,3
23	17,9	19,2	19	23	77	30%	2,4	115	3	12,3	7,1	46,4	10,6	6,1
24	19,8	20,9	53	12	55	21%	3,6	133	3	4,7	5,0	34,2	5,5	5,8
25	22,5	30,0	44	15	55	28%	2,4	142	1	16,0	14,0	35,4	18,1	15,8
26	20,9	19,6	51	16	68	23%	2,3	138	1	8,7	7,9	28,5	12,2	11,1
27	25,0	18,8	42	14	68	21%	2,8	132	1	30,5	18,4	37,5	32,5	19,6
28	22,4	18,8	6	10	81	12%	4,5	113	1	57,9	36,2	76,3	30,4	19,0

**Mean**	**22,8**	**25,6**	**27**	**16**	**74**	**22%**	**3,2**	**134**	**1**	**29,8**	**23,5**	**47,2**	**24,5**	**20,9**
**SD**	**2,5**	**6,8**	**14**	**7**	**12**	**10%**	**0,9**	**10**	**1**	**19,2**	**13,6**	**16,9**	**12,0**	**13,1**
Median	***22,5***	***23,6***	***23***	***15***	***76***	***21%***	***3,3***	***133***	***1***	***26,9***	***23,1***	***47,7***	***25,4***	***19,6***
